# Δ^9^-Tetrahydrocannabinol During Adolescence Attenuates Disruption of Dopamine Function Induced in Rats by Maternal Immune Activation

**DOI:** 10.3389/fnbeh.2019.00202

**Published:** 2019-09-06

**Authors:** Salvatore Lecca, Antonio Luchicchi, Maria Scherma, Paola Fadda, Anna Lisa Muntoni, Marco Pistis

**Affiliations:** ^1^Department of Biomedical Sciences, Division of Neuroscience and Clinical Pharmacology, University of Cagliari, Monserrato, Italy; ^2^Section of Cagliari, Neuroscience Institute, National Research Council of Italy (CNR), Monserrato, Italy

**Keywords:** dopamine neurons, maternal immune activation, cannabinoids, adolescence, electrophysiology, schizophrenia

## Abstract

The combination of prenatal, such as maternal infections, and postnatal environmental insults (e.g., adolescent drug abuse) increases risks for psychosis, as predicted by the two-hit hypothesis of schizophrenia. Cannabis abuse during adolescence is widespread and is associated with increased risk of psychoses later in life. Here, we hypothesized that adolescent Δ^9^-tetrahydrocannabinol (THC) worsens the impact of prenatal maternal immune activation (MIA) on ventral tegmental area (VTA) dopamine cells in rat offspring. Additionally, since substance abuse disorder is particularly prevalent among schizophrenia patients, we also tested how VTA dopamine neurons in MIA offspring respond to acute nicotine and cocaine administration. We used a model of neurodevelopmental disruption based on prenatal administration of the polyriboinosinic-polyribocytidilic acid [poly (I:C)] in rats, which activates the maternal immune system by mimicking a viral infection and induces behavioral abnormalities and disruption of dopamine transmission relevant to psychiatric disorders in the offspring. Male offspring were administered THC (or vehicle) during adolescence (PND 45–55). Once adult (PND 70–90), we recorded the spontaneous activity of dopamine neurons in the VTA and their responses to nicotine and cocaine. MIA male offspring displayed reduced number, firing rate and altered activity pattern of VTA dopamine cells. Adolescent THC attenuated several MIA-induced effects. Both prenatal [poly (I:C)] and postnatal (THC) treatments affected the response to nicotine but not to cocaine. Contrary to our expectations, adolescent THC did not worsen MIA-induced deficits. Results indicate that the impact of cannabinoids in psychosis models is complex.

## Introduction

Environmental factors, such as prenatal exposure to a variety of infectious agents and consequent maternal immune activation (MIA), can lead to aberrant brain development, emerging in pathological phenotypes, such as autism and schizophrenia (Hornig et al., 2018). An association between MIA and increased risks of developing psychiatric disorders in offspring later in life has been reported by preclinical investigations and epidemiological studies in humans (Meyer et al., 2011).

*In utero* exposure to polyriboinosinic-polyribocytidylic acid (Poly I:C), a double-stranded synthetic RNA that activates an innate immune response, induces MIA in rodents by mimicking a viral infection and has been shown to induce schizophrenia- or autism-like phenotypes in rodents (Zuckerman et al., 2003). Hence, offspring display behavioral abnormalities, e.g., impairment in recognition memory, in social interactions and in sensorimotor gating as well as alterations in brain regions key in the neuropathology of psychoses, such as the dopaminergic ventral tegmental area (VTA; Patterson, 2002, 2009; Meyer et al., 2005; Boksa, 2010). Indeed, previous studies reported an increase in the number of TH-immunoreactive neurons in the VTA, TH-positive terminals in the striatum (Meyer et al., 2008; Winter et al., 2009; Vuillermot et al., 2010), increases in evoked striatal dopamine release *ex vivo* (Zuckerman et al., 2003) and enhanced dopamine levels in the prefrontal cortex and lateral globus pallidus (Winter et al., 2009). In our previous studies we observed a marked alteration of VTA dopamine neuron activity (reduced firing rate, reduced number of spontaneously active cells and altered firing pattern) in male but not female offspring coupled with disruption of sensorimotor gating and of cognitive and social behavior, and increase in dopamine levels in the nucleus accumbens (Luchicchi et al., 2016; De Felice et al., 2019).

Besides the prenatal period, adolescence is also a critical window of enhanced vulnerability. During adolescence the brain is particularly susceptible to perturbations, such as exposure to drugs of abuse, which can disrupt cognitive, emotional, and social maturation (Crews et al., 2007). Cannabis is the most widely used illegal drug during adolescence and its consumption might induce neurobiological changes that affect adult brain function (Rubino and Parolaro, 2016).

The dopamine system is particularly sensitive to cannabinoids. Both Δ^9^-tetrahydrocannabinol (THC) and synthetic cannabinoids induce increases in firing rate of mesolimbic and mesocortical VTA dopamine cells (Diana et al., 1998; Gessa et al., 1998) and in extracellular dopamine levels in terminal regions (Tanda et al., 1997). Accordingly, in humans, THC reduces [^11^C]raclopride binding in the ventral striatum, consistent with a modest increase in dopamine release (Bossong et al., 2009, 2015) and exacerbates psychotic symptoms (Mason et al., 2009). We and others reported that adolescent THC administration induced long-lasting changes in the response to dopamine cells to drugs of abuse and enhanced behavioral responses and self-administration (Pistis et al., 2004; Scherma et al., 2016) which might extend across generations (Vassoler et al., 2013). Moreover, adolescent administration of cannabinoids is associated with schizophrenia-like deficits in adult rodents (Rubino et al., 2009; Leweke and Schneider, 2011).

Considering that in humans early marijuana intake is associated with increased risk of psychoses later in life (Arseneault et al., 2004; Fergusson, 2004; Degenhardt and Hall, 2006), our hypothesis is that cannabinoid administration during adolescence in male rats exposed to MIA would worsen the outcome, as the two-hits hypothesis of schizophrenia (genetic/prenatal plus postnatal environment factors) predicts. Additionally, since substance abuse disorder, specifically heavy tobacco smoking (Winterer, 2010) and stimulant use disorder (Hunt et al., 2018), is particularly prevalent among schizophrenia patients we also tested how VTA dopamine neurons in MIA offspring treated with THC and their controls respond to acute nicotine and cocaine administration.

## Materials and Methods

All procedures were performed in accordance with the European legislation EU Directive 2010/63 and were approved by the Animal Ethics Committee of the University of Cagliari and by Italian Ministry of Health (auth. n. 658/2015-PR). Animals were housed in groups of three to six in standard conditions of temperature (21 ± 1°C) and humidity (60%) under a 12 h light/dark cycle (lights on at 7:00 A.M.) with food and water available *ad libitum*. We made all efforts to minimize animal discomfort and to reduce the number of animals used.

### Prenatal Treatment

Female Sprague–Dawley rats (Envigo, Italy) were mated at the age of 3 months. The first day after the copulation was defined as gestational day 1 (GD 1). MIA was induced at GD 15, following the procedure described by Zuckerman et al. (2003). Dams were anesthetized with isoflurane 2% and a single dose of Poly I:C (4.0 mg/kg, i.v.; InvivoGen, San Diego, CA, USA) or an equivalent volume of endotoxin-free saline solution was administered in the lateral vein of the tail. To assess the efficacy of Poly I:C injection, all pregnant rats were weighed for the first 3 days after the administration of either Poly I:C or saline to evaluate weight loss as underlined by previous investigations (Zuckerman et al., 2003; Wolff and Bilkey, 2010). After weaning, male offspring were housed with littermates and maintained undisturbed until adolescent treatment (PND 45–55) and experiments in adulthood (PND 70–90). Male rats were randomly assigned to the experimental procedures and care was taken to avoid assigning more than three animals from the same litter to the same experimental group (Kentner et al., 2019).

### Adolescent Treatment

Male rats were intraperitoneally injected with THC (THC-Pharm GmbH) or vehicle (1% ethanol, 2% Tween 80 and saline) at PND 45, in the mid-adolescence period. Increasing doses of THC (2.5 mg/kg, PND 45–47; 5 mg/kg, PND 48–51; 10 mg/kg, PND 52–55) or vehicle were given twice/day for 11 consecutive days. Theses doses of THC were chosen according to the literature (Scherma et al., 2016). Body weight and food intake were monitored for the entire period of treatment.

### *In vivo* Electrophysiological Experiments

*In vivo* electrophysiology experiments were carried out at PND 70–90. This age window, which corresponds to the young adulthood in humans, was selected as it is the most vulnerable age for the onset of schizophrenia (Häfner, 2003). Moreover, studies on the ontogeny of MIA-induced deficits showed that these are evident at PND 70 (Romero et al., 2010; Vuillermot et al., 2010).

*In vivo* electrophysiological recordings were performed as described previously (Melis et al., 2008, 2009; Luchicchi et al., 2016). At PND 70–90, male rats were anesthetized with urethane (1.3 g/kg, i.p.) and placed in the stereotaxic apparatus (Kopf, Tujunga, CA, USA) with their body temperature maintained at 37 ± 1°C by a heating pad.

For the placement of a recording electrode, the scalp was retracted, and one burr hole was drilled above the parabrachial pigmented nucleus (PBP) of the posterior VTA (AP, 5.8–6.2 mm posterior from Bregma, L, 0.4–0.6 mm lateral from midline) according to the Atlas of Rat Brain (Paxinos and Watson, 2007). We selected this subregion as it contains the largest density of dopamine cells as compared to the more medial portions of the posterior VTA.

Extracellular single-unit activity of dopamine neurons located in the VTA (V, 7.0–8.0 mm from the cortical surface) was recorded with glass micropipettes filled with 2% Pontamine sky blue (PSB) dissolved in 0.5 M sodium acetate (impedance 2.5–5 MΩ). The population spontaneous activity of VTA dopamine cells was determined in 6–9 predetermined tracks separated by 200 μm each other. Putative VTA dopamine neurons were selected when all criteria for identification were fulfilled: firing rate <10 Hz and duration of action potential >2.5 ms as measured from start to end (Grace and Bunney, 1983). At the end of the experimental session, inhibition of spontaneous activity by dopamine receptor agonists and subsequent reversal by dopamine receptor antagonists was tested. Bursts were defined as the occurrence of two spikes at interspike interval <80 ms, and terminated when the interspike interval exceeded 160 ms (Grace and Bunney, 1984). The electrical activity of each neuron was recorded for 2–3 min. Single-unit activity was filtered (bandpass 0.1–10,000 Hz) and individual action potentials were isolated and amplified (Neurolog System, Digitimer, Hertfordshire, UK), displayed on a digital storage oscilloscope (TDS 3012, Tektronics, Marlow, UK) and digitally recorded. Experiments were sampled on-line and off-line with Spike2 software (Cambridge Electronic Design, Cambridge, UK) by a computer connected to CED 1401 interface (Cambridge Electronic Design, Cambridge, UK). At the end of recording sessions, DC current (15 mA for 5 min) was passed through the recording micropipette in order to eject PSB for marking the recording site. Brains were then rapidly removed and frozen in isopentane cooled to −40°C. The position of the electrodes was microscopically identified on serial 60 μm sections stained with Neutral Red.

In separate experiments where the effects of nicotine and cocaine were assessed, after 5 min of stable baseline activity, cocaine (Akzo Pharma Division Diosynth, Oss, Netherlands) was administered i.v. at exponentially increasing cumulative doses (0.25–2 mg/kg) every 2 min or nicotine [(-)-nicotine hydrogen tartrate), Sigma-Aldrich, Italy] at a bolus dose of 0.2 mg/kg.

### Statistical Analysis

Averaged data from different experiments are given as mean ± SEM. Data were checked for outliers (ROUT test) and statistical significance was assessed using Student’s *t*-test, one-way ANOVA, two-way ANOVA and two-way ANCOVA, where appropriate. *Post hoc* multiple comparisons were made using the Sidak’s test. Data were analyzed using GraphPad Prism (San Diego, CA, USA). The significance level was established at *P* < 0.05.

## Results

In agreement with previous studies (Zuckerman et al., 2003), rat dams underwent a significant weight loss in the 24 h following Poly I:C systemic administration (−4.9 ± 2.8 g *n* = 8; vs. controls +7.5 ± 2.4 g *n* = 7; *P* < 0.01, Student’s *t*-test; data not shown). This weight loss indicates that Poly I:C treatment induced a flu-like syndrome in treated rats (Kentner et al., 2019). However, Poly I:C treatment did not affect litter size (controls: 11.6 ± 1.8 pups, *n* = 8; Poly I:C: 12.4 ± 1.2 pups, *n* = 7, *P* = 0.72, Student’s *t*-test). As our previous studies determined that detrimental effects induced by MIA were only evident in males (De Felice et al., 2019), we evaluated the effect of pubertal THC solely in male rats. Hence, prenatal Poly I:C or vehicle male offspring were randomly assigned to the adolescent THC or vehicle groups, taking care that no more than three animals from the same litter were assigned to the same experimental group or procedure (Kentner et al., 2019). Therefore, electrophysiological experiments were carried out in four experimental groups: vehicle-vehicle, vehicle-THC, Poly I:C-vehicle and Poly I:C-THC.

We next determined if Poly I:C prenatal and THC postnatal treatments affect spontaneous activity of dopamine cells, by carrying out a population sample in the VTA. For these experiments we utilized *n* = 14 vehicle-vehicle (from 6 litters), *n* = 19 Poly I:C-vehicle (from 8 litters), *n* = 8 vehicle-THC (from 4 litters) and *n* = 10 Poly I:C-THC (from 5 litters) male offspring.

**Figure 1:**
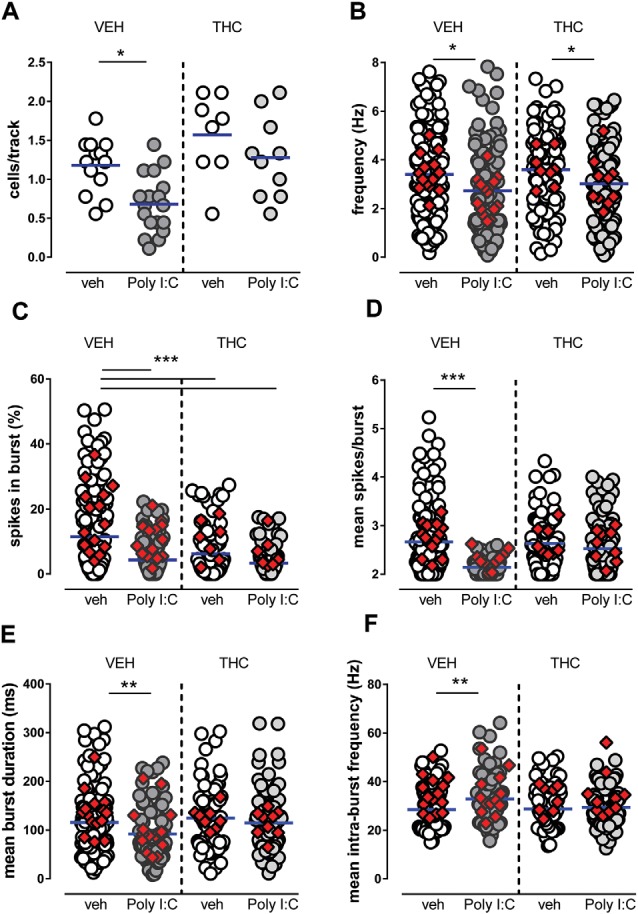
Effects of maternal immune activation (MIA) and adolescent Δ^9^-tetrahydrocannabinol (THC) administration on ventral tegmental area (VTA) dopamine neuron activity *in vivo*. Adolescent THC administration prevented the Poly I:C-induced decrease in the number of spontaneously active VTA dopamine neurons **(A)** but not the decrease in firing rate **(B)**. Graphs show the effect of poly IC and THC (or vehicles) in the percentage of spikes in burst **(C)**, mean burst duration **(D)**, mean number of spikes in bursts **(E)** and intra-burst frequency **(F)**. Superimposed colored diamonds show the averages for each individual rat. Both Poly I:C and THC, or their combination, induced a reduction in the percentage of spikes in bursts **(C)**, whereas THC prevented alterations induced by Poly I:C in the other electrophysiological parameters **(D,E)**. The number of cells for each group is: veh-veh, *n* = 156; Poly I:C-veh, *n* = 121; veh-THC, *n* = 101; Poly I:C-THC, *n* = 117. The horizontal blue line represents the mean. Statistical analysis was conducted with two-way ANOVA (Poly I:C and THC treatments as factors) and Sidak’s multiple comparison test. Asterisks on graphs represent the result of the Sidak’s multiple comparison test: *P < 0.05, **P < 0.01, ***P < 0.001.

The number of cells/track (Figure 1A), which is an index of population activity of dopamine neurons in the VTA, was significantly reduced by Poly I:C treatment in vehicle-treated but not in THC-treated male offspring [two-way ANOVA: effect of Poly I:C, *F*_(1,45)_ = 9.49, *P* < 0.01; effect of THC, *F*_(1,45)_ = 14.78, *P* < 0.001; interaction between treatments, *F*_(1,45)_ = 0.66, *P* > 0.05; *post hoc* Sidak’s test: significant effect only between vehicle-vehicle and Poly I:C-vehicle rats (*t*_(45)_ = 3.2, *P* < 0.05, Figure 1A)]. The firing rate was reduced by Poly I:C treatment in both vehicle- and THC-treated rats (Figure 1B; two-way ANOVA: effect of Poly I:C, *F*_(1,454)_ = 13.53, *P* < 0.01; effect of THC, *F*_(1,454)_ = 1.98, *P* > 0.05; interaction between treatments, *F*_(1,454)_ = 0.10, *P* > 0.05). The percentage of spikes per burst was reduced by both Poly I:C and THC treatments (Figure 1C) [two-way ANOVA: effect of Poly I:C, *F*_(1,386)_ = 26.28, *P* < 0.001; effect of THC, *F*_(1,386)_ = 9.92, *P* < 0.01; interaction between treatments, *F*_(1,386)_ = 4.67, *P* < 0.05; *post hoc* Sidak’s test: significant effect between vehicle-vehicle and all other groups: (*t*_(386)_ = 5.5, *t*_(386)_ = 3.9, *t*_(386)_ = 6.1 for vehicle-vehicle vs. Poly I:C-vehicle, vehicle-THC, Poly I:C-THC, respectively, *P* < 0.001 for all comparisons, Figure 1C)]. The number of spikes per burst (Figure 1D) was significantly reduced by Poly I:C treatment only in vehicle-treated rats [two-way ANOVA: effect of Poly I:C, *F*_(1,334)_ = 20.85, *P* < 0.0001; effect of THC, *F*_(1,334)_ = 6.32, *P* < 0.05; interaction between treatments, *F*_(1,334)_ = 9.30, *P* < 0.01; *post hoc* Sidak’s test: significant effect only between vehicle-vehicle and Poly I:C-vehicle rats (*t*_(334)_ = 5.4, *P* < 0.0001, Figure 1D)]. The mean burst duration (Figure 1E) was also significantly reduced by Poly I:C treatment in vehicle-treated but not in THC-treated rats [two-way ANOVA: effect of Poly I:C, *F*_(1,367)_ = 3.58, *P* > 0.05; effect of THC, *F*_(1,367)_ = 3.42, *P* > 0.05; interaction between treatments, *F*_(1,367)_ = 5.62, *P* < 0.05; *post hoc* Sidak’s test: significant effect only between vehicle-vehicle and Poly I:C-vehicle rats (*t*_(367)_ = 3.2, *P* < 0.01, Figure 1E)]. Similarly, the mean intraburst frequency (Figure 1F) was significantly reduced by Poly I:C treatment in vehicle-treated but not in THC-treated offspring [two-way ANOVA: effect of Poly I:C, *F*_(1,338)_ = 7.87, *P* < 0.01; effect of THC, *F*_(1,338)_ = 3.26, *P* > 0.05; interaction between treatments, *F*_(1,338)_ = 4.25, *P* < 0.05; *post hoc* Sidak’s test: significant effect only between vehicle-vehicle and Poly I:C-vehicle rats (*t*_(338)_ = 3.6, *P* < 0.01, Figure 1F)].

In summary, in Poly I:C-vehicle male offspring we detected a reduced number of spontaneously active cells, lower frequency, shorter bursts, a lower number of action potentials per burst, when compared with vehicle-vehicle offspring. Adolescent THC treatment in vehicle-THC rats did not exert significant effects, except for the percentage of spikes in burst, which was reduced when compared to vehicle-vehicle offspring, whereas in Poly I:C-THC offspring, THC reversed the effects of MIA on cells/track index, mean spikes/burst, mean burst duration and mean intra-burst frequency. Our data indicate that dopamine cells in prepubertal THC-treated offspring are less affected by MIA when compared with Poly I:C-vehicle rats.

Considering that we recorded several neurons from each individual rat and that each cell was considered as an independent replicate, a two-way ANCOVA was carried out with treatments as factors and individual subjects as covariate, to exclude that differences among individual rats had significant effects. The results indicated that individual subjects had no significant effect overall (two-way ANCOVA *P* > 0.05 for all parameters).

We next examined the response of VTA dopamine cells to a nicotine challenge and to cumulative doses of cocaine.

**Figure 2:**
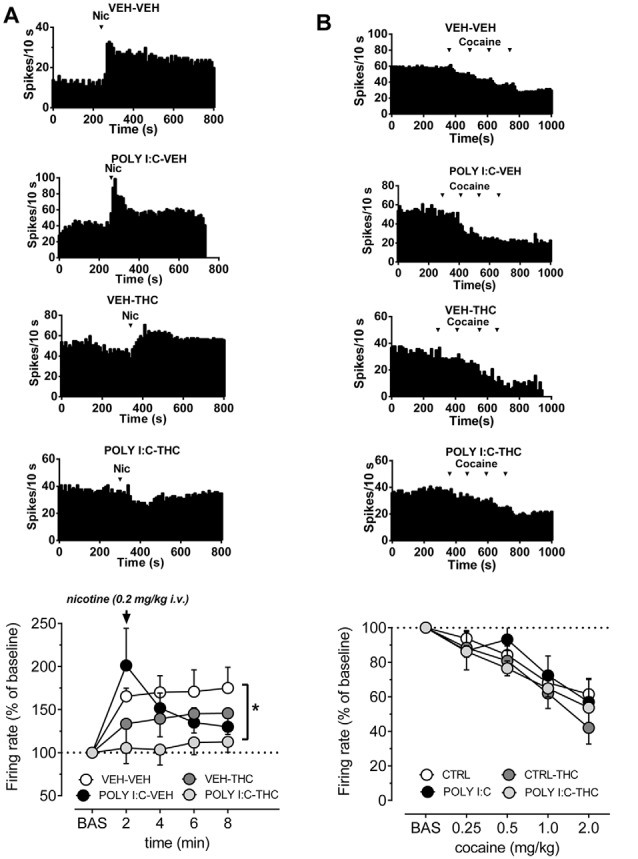
Effects of nicotine and cocaine on firing rate of VTA dopamine neurons in prenatal Poly I:C and adolescent THC treated offspring and their controls. **(A)** Representative firing rate histograms of VTA dopamine neurons recorded from vehicle-vehicle, Poly I:C-vehicle, vehicle-THC and Poly I:C-THC rats showing the effects of a bolus dose of nicotine (0.2 mg/kg, i.v.). Arrows indicate the times of nicotine injection. The graph shows that the combination of prenatal Poly I:C and adolescent THC prevented nicotine-induced increase of firing rate (vehicle-vehicle, *n* = 8; Poly I:C-vehicle, *n* = 6; vehicle-THC, *n* = 6 and Poly I:C-THC, *n* = 6; two-way ANOVA and Sidak’s test, *P < 0.05). **(B)** Representative firing rate histograms of VTA dopamine neurons recorded from vehicle-vehicle, Poly I:C-vehicle, vehicle-THC and Poly I:C-THC rats showing the effects of cumulative doses of cocaine (0.25–2.0 mg/kg, i.v.). Arrows indicate the times of cocaine injections (0.25, 0.25, 0.5, 1.0 mg/kg). The bottom graph displays the dose–response curves of the effect of cumulative doses of cocaine on the firing rate of VTA DA neurons recorded from vehicle-vehicle (*n* = 5), Poly I:C-vehicle (*n* = 6), vehicle THC (*n* = 4) and Poly I:C-THC (*n* = 4). Results are presented as mean ± SEM of firing rate expressed as a percentage of baseline levels.

Figure 2A shows that pre- and postnatal treatments affect the response of VTA dopamine cells to nicotine (0.2 mg/kg, i.v.). The dose of nicotine was selected as it approximately corresponds to the i.p. dose of nicotine (0.4 mg/kg) that induces a robust conditioned place preference and, consistently, induces also a strong increase in firing rate of VTA dopamine cells in control animals (Melis et al., 2008; Mascia et al., 2011; Sagheddu et al., 2019). Spontaneous activity of VTA neurons was recorded for 5 min then a bolus dose of nicotine was injected intravenously. In vehicle-vehicle rats nicotine induced a robust increase in firing rate, amounting to ~165% of baseline (*F*_(4,7)_ = 6.7, *P* < 0.05, one-way ANOVA), which remained stable across the recording time. On the other hand, nicotine did not significantly affect firing rate of VTA cells either in Poly I:C-vehicle, vehicle-THC nor in Poly I:C-THC rats (*F*_(4,5)_ = 3.6, *F*_(4,5)_ = 2.8, *F*_(4,5)_ = 0.4, respectively, *P* > 0.05, one-way ANOVA for all comparisons). When curves were compared across groups, two-way ANOVA revealed a significant interaction between factors (time and treatments; *F*_(12,88)_ = 2.10, *P* < 0.05) and *post hoc* analysis indicates that nicotine-induced effects were significantly different in Poly I:C-THC rats when compared to the vehicle-vehicle group (*t*_(110)_ = 2.5, *P* < 0.05, Sidak’s multiple comparison test).

It is well established that cocaine inhibits dopamine neurons *via* increased somatodendritic dopamine release acting on D2 autoreceptors (Einhorn et al., 1988). As illustrated in Figure 2B, we confirmed that cocaine (0.25, 0.5, 1.0 and 2.0 mg/kg, i.v., expressed as the final cumulative doses at each point, as we injected 0.25, 0.25, 0.5 and 1 mg/kg, i.v.), dose-dependently reduced firing rate of dopamine cell to approximately 50% in vehicle-vehicle rats (*F*_(4,5)_ = 17.29, *P* < 0.001, one-way ANOVA). This inhibitory effect was similar to the control group and statistically significant also in Poly I:C-vehicle (*F*_(4,5)_ = 4.5, *P* < 0.05, one-way ANOVA), vehicle-THC (*F*_(4,3)_ = 29.9, *P* < 0.01, one-way ANOVA) and Poly I:C-THC rats (*F*_(4,3)_ = 81.6, *P* < 0.01, one-way ANOVA). The comparison across groups revealed that neither Poly I:C nor THC treatments, or their interaction with the doses of cocaine, changed the inhibitory effect of cumulative doses of cocaine onto VTA dopamine cells (*F*_(12,64)_ = 0.63, *P* = 0.8, two-way ANOVA).

## Discussion

The present findings confirm our previous studies that MIA, evoked by maternal exposure to Poly I:C, induces harmful effects in offspring, namely disruption of dopamine cell electrophysiological activity: (i) reduced number of spontaneously active cells; (ii) decrease in their firing rate; and (iii) profound alterations in their firing pattern (Luchicchi et al., 2016; De Felice et al., 2019). We and other groups showed that changes in dopamine transmission translate into abnormal behavior such as disrupted sensorimotor gating, deficits in cognition and social interactions (Zuckerman et al., 2003; Meyer et al., 2011; Luchicchi et al., 2016). The risk to develop schizophrenia has often been hypothesized with models requiring two hits in order to induce the clinical phenotype: an early priming in a genetically/prenatally predisposed individual and a second, likely environmental, insult (Davis et al., 2016). Consistent with this scenario, combining exposure to prenatal immune challenge and peripubertal stress in mice was shown to induce synergistic pathological effects on adult behavior and neurochemistry (Giovanoli et al., 2013, 2016).

Cannabis exposure during adolescence is consistently associated with an increased risk to develop schizophrenia later in life and with an earlier onset of the disease (Arseneault et al., 2004; Fergusson, 2004; Degenhardt and Hall, 2006). Preclinical findings consistently indicate that adolescent cannabinoid agonist intake induces long-term behavioral impairment and depressive-like signs (Rubino et al., 2009; Rubino and Parolaro, 2016). Therefore, it may represent a risk factor for developing psychotic-like symptoms in adulthood (Rubino et al., 2008).

Thus, our hypothesis was that exposure to THC during adolescence might exacerbate the disruption in VTA dopamine cell activity observed in offspring following MIA. Contrary to our expectations, adolescent THC did not induce effects in prenatal vehicle-treated animals, apart from a decrease in the bursting activity of dopamine cells, whereas in Poly I:C-treated offspring it attenuated several alterations induced by MIA. Notably, MIA with Poly I:C was shown to induce in rats persistent increases in cannabinoid CB1 receptor expression in adulthood in sensory cortex and hypothalamus assessed by PET (Verdurand et al., 2014). These findings indicate that prenatal Poly I:C leads to region-specific long-term alterations in the integrity of the endocannabinoid system that mirror those observed in patients with schizophrenia in post-mortem and *in vivo* PET studies (Köfalvi and Fritzsche, 2008). It is tempting to speculate that THC in adolescence might induce changes in CB1 receptor expression that, in our model, counteract those induced by MIA. As an example, in MIA-exposed male offspring we observed a decrease in the probability of glutamate and GABA release onto dopamine cells, indexed by an increase in the paired-pulse ratio of excitatory and inhibitory currents coupled with a reduced frequency of miniature inhibitory and excitatory postsynaptic currents (De Felice et al., 2019). As the release of GABA and glutamate is tightly regulated by 2-arachidonoylglicerol (2-AG) acting on presynaptic CB1 receptors (Melis et al., 2004, 2014), we can speculate that this reduced neurotransmitter release might be caused by an increased expression or activity of CB1 receptors on GABA or glutamate terminals, consistent with the study by Verdurand et al. (2014). Alternatively, one possibility is that of an enhanced biosynthesis of 2-AG by DAG-lipase in dopamine cells or reduced degradation by MAG-lipase. How adolescent THC might reverse these changes is not known. One intriguing possibility is that adolescent subchronic THC might induce a long-lasting tolerance by reducing expression or activity of CB1 receptors, as shown in our previous studies (Pistis et al., 2004; Dudok et al., 2015).

To the best of our knowledge, this is the very first study carried out in a neurodevelopmental schizophrenia model with the phytocannabinoid THC and not with synthetic cannabinoids (Gomes et al., 2014; Aguilar et al., 2018). Interestingly, in line with our findings, the study by Gomes et al. (2014) reported that administration of the synthetic cannabinoid WIN55212 during adolescence did not exacerbate the behavioral and electrophysiological changes in methylazoxymethanol acetate (MAM)-treated rats but attenuated the enhanced locomotor response to amphetamine. On the other hand, in the study by Aguilar et al. (2018), pubertal exposure to WIN55212 or to the fatty acid amide hydrolase (FAAH) inhibitor URB597, which increases endogenous anandamide levels, augmented the proportion of second-generation MAM rats that develop schizophrenia-like deficits. In both studies, the synthetic cannabinoid treatment was able to increase the number of spontaneously active dopamine cells in vehicle-treated animals. Although we observed a trend toward an increase in the cell/track index in vehicle-THC offspring (Figure 1A), this effect did not reach statistical significance. These divergent results with our study might be due to different pharmacology of the cannabinoid agonists used (full vs. partial agonist), to the different length and protocol of adolescent cannabinoid treatment (11 vs. 25 days, continuous vs. intermittent), or to different neurodevelopmental models (MAM vs. MIA).

Epidemiological studies confirm that schizophrenia patients show enhanced prevalence of substance use disorders, particularly concerning nicotine dependence, psychostimulant and cannabis abuse (Kalman et al., 2005; Swendsen et al., 2010). In animal models of psychiatric disorders, responses to psychostimulant or nicotine is altered: locomotor response to psychostimulants is enhanced in neurodevelopmental models of schizophrenia (Gomes et al., 2014; Aguilar et al., 2018), whereas nicotine is more self-administered and ameliorated cognitive deficits in a lipopolysaccharide MIA model of schizophrenia (Waterhouse et al., 2018). Here we tested if prenatal and/or postnatal treatments affected responses of VTA dopamine neurons to nicotine and cocaine. We found a blunted effect of nicotine on VTA dopamine cells in all groups when compared to vehicle-vehicle animals, although this difference reached a statistical significance only in Poly I:C-THC offspring. These results suggest that adolescent THC and MIA, or the combination of both factors, induce persistent changes in neuronal response to nicotine. The reason for this effect requires further investigation. It can be speculated that a reduced response to nicotine in both THC- or MIA-exposed rats might be relevant for the high prevalence of heavy tobacco smoking reported in both cannabis abusers or schizophrenia patients (Kalman et al., 2005; Swendsen et al., 2010), as higher nicotine doses might be required to attain positive subjective effects. On the other hand, the inhibitory effect of cocaine did not change among the four experimental groups.

Our results, together with other previous studies, confirm that the effects of adolescent cannabinoid exposure in MIA-exposed individuals are more complex than expected and that the combination of prenatal and postnatal insults (the double hit hypothesis of schizophrenia) in neurodevelopmental models of schizophrenia needs to be further explored.

## Data Availability

The datasets generated for this study are available on request to the corresponding author.

## Ethics Statement

The animal study was reviewed and approved by Animal Ethics Committee of the University of Cagliari and by Italian Ministry of Health (auth. n. 658/2015-PR).

## Author Contributions

AL and SL contributed to the acquisition of animal data, performed data analysis, contributed to interpretation of results and provided critical revision of the manuscript. MS and PF assisted with acquisition of animal data, analysis and interpretation of findings. PF and AM provided critical revision of the manuscript for important intellectual content. MP was responsible for the study concept and design and drafted the manuscript. All authors critically reviewed the content and approved the final version for publication.

## Conflict of Interest Statement

The authors declare that the research was conducted in the absence of any commercial or financial relationships that could be construed as a potential conflict of interest.
